# From periodontal inflammation to hepatic immune remodeling: emerging concepts of myeloid reprogramming in stage-dependent MASLD

**DOI:** 10.3389/fimmu.2026.2003684

**Published:** 2026-10-22

**Authors:** Xudong Song, Congxiao Zhang

**Affiliations:** Department of Stomatology, the First Hospital of Jilin University, Changchun, China

**Keywords:** cross-organ immune crosstalk, hematopoietic stem and progenitor cells, MASH, MASLD, myeloid remodeling, neutrophil extracellular traps, periodontitis, trained immunity

## Abstract

Periodontitis and metabolic dysfunction-associated steatotic liver disease (MASLD) are common chronic inflammatory disorders. Recent population studies support epidemiological associations between periodontitis and MASLD and related hepatic phenotypes, but substantial heterogeneity remains in their strength and independence, and current evidence is insufficient to establish causality. This review focuses on the periodontal-to-liver direction, using circulating myeloid cells as a central line of inquiry to examine the potential link between periodontitis-associated systemic myeloid reprogramming and intrahepatic myeloid remodeling in MASLD. Direct evidence that periodontitis-conditioned myeloid cells enter the MASLD liver and alter immune remodeling or disease progression is still lacking. Although both processes are independently supported, the cross-organ bridge between them remains unresolved. Evidence is also asymmetric across myeloid pathways: the neutrophil–NET axis has relatively direct functional support at both ends, whereas the monocyte–macrophage axis is supported mainly on the hepatic side. We propose that periodontitis may alter the pre-hepatic functional state of myeloid cells, while their biological effects are expected to depend on the stage and local immune environment of the recipient liver. Testing this model will require lineage-informed cell tracking, stage-specific perturbation, and longitudinal studies integrating periodontal inflammation, circulating myeloid phenotypes, and hepatic phenotypes within the same patients.

## 1 Introduction

### 1.1 Clinical co-occurrence and the question of causality

Periodontitis is a chronic, multifactorial inflammatory disease driven by dysbiotic dental biofilms and dysregulated host immune responses, leading to irreversible destruction of tooth-supporting tissues, including the periodontal attachment and alveolar bone (1–3). In 2023, an international multisociety consensus replaced the term nonalcoholic fatty liver disease (NAFLD) with metabolic dysfunction-associated steatotic liver disease (MASLD), defined as hepatic steatosis in the presence of at least one cardiometabolic risk factor (4); metabolic dysfunction-associated steatohepatitis (MASH) similarly replaced nonalcoholic steatohepatitis (NASH) and describes a progressive phenotype characterized by steatohepatitis and hepatocellular injury (4, 5). Severe periodontitis affected an estimated 1.09 billion people worldwide in 2023 (95% uncertainty interval [UI], 0.915–1.26 billion), representing a 5.73% increase in prevalent cases since 2019 (95% UI, 4.57%–6.91%) (6); according to the Global Burden of Disease Study 2023, approximately 1.3 billion people were living with MASLD (95% UI, 1.2–1.4 billion; 16.1% of the global population) (7). Given the burden of both diseases, whether and how they are biologically connected constitutes an important public health question.

Periodontitis and MASLD share metabolic, host-related, and lifestyle risk factors—including obesity, type 2 diabetes, insulin resistance, and smoking—and both diseases feature chronic low-grade inflammation and dysregulated immune responses (5, 8–10). Their epidemiological co-occurrence may therefore reflect these shared risk factors rather than a direct effect of periodontitis, and periodontitis cannot be assumed to be an independent cause of MASLD. Observational studies have reported associations between periodontal disease and incident or prevalent fatty liver disease, but the magnitude and consistency of these associations vary with study design, disease definition, and adjustment for metabolic and lifestyle confounders (9, 11–13). Findings from Mendelian randomization studies are likewise inconsistent, leaving the causal direction of the periodontal–hepatic relationship uncertain (14–16). Irrespective of causality, MASLD spans a continuous clinicopathologic spectrum from isolated hepatic steatosis to MASH, progressive fibrosis, cirrhosis, and hepatocellular carcinoma; this heterogeneity further complicates mechanistic interpretation (5, 17–19).

### 1.2 Epidemiological evidence: associations, bidirectionality and unresolved causality

Recent population-based studies and meta-analyses report associations between periodontitis and MASLD/NAFLD, with pooled estimates ranging from a positive association (OR 1.34) to a null association (OR 1.04), depending on the periodontal definition used (13, 20, 21). Longitudinal cohorts suggest that periodontitis may be associated with the development of fatty liver and with progression of chronic liver disease, particularly in metabolically susceptible populations (11, 12, 22, 23); cross-sectional studies have likewise reported associations between periodontal status and steatosis or its surrogate indices (24, 25). Longitudinal data suggest bidirectionality rather than a single pathway: MASLD/NAFLD scores have been associated with subsequent incident periodontitis (26, 27), whereas poorer self-reported oral health has been associated with incident MASLD (28). Reported effect sizes are generally small, however, and vary with the definition of periodontitis, the hepatic outcome measure, and adjustment for metabolic confounders; Mendelian randomization studies have reached similarly inconsistent conclusions (14–16). Overall, the current human evidence supports clinical co-occurrence and a possible bidirectional temporal association but does not establish an independent causal role for either disease in the development or progression of the other; **Table 1** summarizes the design, population, periodontal definition, hepatic outcome, and effect estimates of these studies. Interventional evidence remains preliminary: one small randomized trial suggested that intensive periodontal treatment may reduce alanine aminotransferase (ALT) levels and *Porphyromonas gingivalis* antibody titers in patients with NAFLD and periodontitis (29).

**Table 1:** Human studies linking periodontitis to steatotic liver disease.

Study	Design & population	Periodontitis definition	Liver outcome	Adjusted for metabolic factors	Main effect	Key limitation
A. Observational association studies						
Akinkugbe 2017 (11)	German SHIP cohort; n=2,623; ~7.7-y follow-up	CAL ≥3 mm and PD ≥4 mm; by % affected sites	Incident NAFLD (ultrasound/ALT)	Partial multivariable adjustment; residual confounding	CAL ≥3 mm in ≥30% sites: IRR 1.60 (95% CI 1.05–2.43); no consistent PD dose-response	Single cohort; outcome misclassification; partial-mouth exam/loss to follow-up
Akinkugbe 2018 (100)	U.S. HCHS/SOL cross-sectional study; n=11,914 Hispanic/Latino adults	CAL ≥3 mm and PD ≥4 mm; 0, <30%, or ≥30% affected sites	Elevated ALT/AST; FLI ≥60	Partial adjustment; associations attenuated after adjustment	Crude PD dose-response; PD and CAL non-significant after adjustment	Cross-sectional; enzyme-based outcome; residual confounding
Alazawi 2017 (97)	NHANES III cross-sectional, n=8,172 + London NAFLD cohort, n=69 (45 biopsies)	NHANES: PPD criteria; London: BPE criteria	Steatosis, NFS, biopsy NASH/F2–F4 fibrosis	NHANES: metabolic adjustment; cohort: mainly diabetes	NHANES: NS after adjustment; cohort: periodontitis 33% vs 3% with significant fibrosis; NASH OR 12.2	Mainly cross-sectional; small clinical cohort; differing definitions/incomplete biopsy
Iwasaki 2018 (25)	Japanese cross-sectional health-check cohort; n=1,226	≥1 tooth with PPD ≥4 mm; severity by PPD	Ultrasound-diagnosed NAFLD	Adjusted for demographic, lifestyle and metabolic factors	PPD ≥4 mm: aOR 1.88 (1.18–2.99); women OR 2.97	Cross-sectional; single center; ultrasound outcome
Weintraub 2019 (24)	NHANES III cross-sectional; n=5,421 U.S. adults	CDC/AAP moderate–severe periodontitis (CAL/PD)	NAFLD by ultrasound, fibrosis score, FLI and US-FLI	Multivariable clinical/sociodemographic adjustment	Association across definitions: OR 1.54 (US), 3.10 (FS), 1.61 (FLI), 2.21 (US-FLI)	Cross-sectional; partial-mouth exam; non-biopsy liver measures
Helenius-Hietala 2019 (23)	Finnish population cohort; n=6,165; ~13-y follow-up	Mild–moderate: 1–4 teeth with PPD ≥4 mm; advanced: ≥5 teeth	Severe liver events (hospitalization, liver cancer, liver-related death)	Extensive metabolic/lifestyle/socioeconomic adjustment	Advanced periodontitis: HR 3.29 (1.53–7.05) in the fully adjusted model (3.69 [1.79–7.60] with minimal adjustment); NAFLD subgroup HR 6.94 (1.43–33.6)	Only 79 events; wide subgroup CIs; composite liver outcome
Kuroe 2021 (22)	5-y Japanese cohort; n=341 NAFL adults without baseline fibrosis	PD/CAL; mean CAL as main measure	Incident fibrosis by NFS ≥−1.455	Multivariable adjustment; obesity-stratified analyses	Overall OR 1.82 (0.94–3.49); obese subgroup OR 2.87 (1.23–6.69)	Small cohort; NFS-based fibrosis; single occupational population
Sato 2022 (98)	Japanese cross-sectional NAFLD cohort; n=164	PPD, CAL and BOP; main comparison by PPD ≥4 mm sites	Fibrosis/liver stiffness by VCTE and MRE	Partial clinical adjustment; metabolic control incomplete	>10 PPD ≥4 mm sites: MRE 3.6 vs 3.0 kPa; OR 2.05 for MRE ≥3.4 kPa	Cross-sectional; small NAFLD-only sample; no biopsy
Shin 2022 (12)	Korean NHIS retrospective cohort; n=165,032; 11-y follow-up	ICD-10 periodontal disease; severity/visit frequency considered	Incident NAFLD (ICD-10)	Broad sociodemographic/lifestyle/comorbidity adjustment	Periodontitis: aHR 1.04 (1.01–1.07); up to 1.14 with 3 visits	Claims-based exposure/outcome; no clinical periodontal or imaging data; residual confounding
Shine 2024 (26)	Nationwide Korean cohort; n=115,619; age ≥40; mean follow-up 7.4 y	Outcome: incident CP by ICD-10 + dental treatment	Exposure: MASLD/MetALD mainly by FLI ≥30 + cardiometabolic criteria	Adjusted for demographics, comorbidity, smoking/exercise; metabolic factors partly in MASLD definition	MASLD aHR 1.14 (1.11–1.17); MetALD 1.21 (1.15–1.27)	Reverse-direction study; claims-based CP; FLI-based steatosis
Joo 2025 (27)	Korean retrospective cohort; n=129,087; mean follow-up 7.4 y	Outcome: incident CP by diagnostic codes + dental procedures	Exposure: NAFLD scores (FLI, HSI, FSI)	Multivariable Cox models; metabolic variables embedded in scores	High FLI HR 1.32 (1.18–1.47); HSI 1.13; FSI 1.23	Reverse-direction study; administrative CP; score-based NAFLD
Liu 2025 (88)	UK Biobank; cross-sectional n=438,905; MRI subset n=39,656; median 10.3 y	Self-reported gum pain/bleeding/loose teeth; ≥1 symptom = PD risk	SLD by FLI; liver fat by MRI-PDFF	Extensive demographic/lifestyle/metabolic adjustment	SLD OR 1.10 (1.08–1.13); PDFF difference 0.091 (0.047–0.139); BMI/CRP/glucose partly mediated	Self-reported PD; healthy-volunteer bias; no baseline PDFF
Ye 2026 (28)	Prospective UK Biobank; n≈478,000; median 13.2 y	OHS/PDR index from 6 self-reported oral symptoms	Incident MASLD; 6,980 cases	Extensive adjustment + mediation/PRS analyses	Poor oral health HR 1.60 (1.42–1.80); PDR HR 1.18 (1.11–1.25); mediation up to 19.4%	Self-reported oral exposure; no clinical periodontitis; residual confounding
Hiroshimaya 2026 (99)	Japanese dental-clinic cross-sectional study; n=2,453; mean age 46.9 y	CPI; ≥1 index tooth PPD ≥4 mm; PPD ≥6 mm/BOP for severity	NAFLD/fibrosis risk by FLI, NFS and FIB-4	Multivariable adjustment; socioeconomic control incomplete	PPD ≥6 mm: NFS >0.675 OR 4.11 (1.06–15.99); FLI ≥60 OR 1.76 (1.12–2.76)	Cross-sectional; surrogate liver scores; small high-risk groups/partial CPI exam
B. Systematic reviews and meta-analyses						
Wijarnpreecha 2020 (20)	Systematic review/meta-analysis; 5 studies; n=27,703	Mainly PPD >3.5–4 mm; some CAL ≥3 mm	Study-specific NAFLD definitions	Pooled crude and adjusted estimates	Adjusted estimates: PPD OR 1.13 (0.95–1.35); CAL OR 1.08 (0.94–1.24), both NS	Few studies; high heterogeneity; variable definitions/adjustment
Xu 2023 (21)	Systematic review/meta-analysis; 7 studies; n=192,815	Variable periodontitis definitions	Variable NAFLD diagnostic methods	Study-specific adjustment strategies	Pooled OR 1.04 (0.97–1.12; I²=58%), NS; cohort studies suggested a small increase	Moderate heterogeneity; few cohorts; variable definitions
Li 2026 (13)	Systematic review/meta-analysis; 11 studies (8 cross-sectional, 3 cohort); n=225,091	Variable definitions, mainly CAL/PPD	Ultrasound, biochemical indices or diagnostic codes	Mainly pooled adjusted estimates; covariates varied	Cross-sectional OR 1.34 (1.13–1.58; I²=68%); CAL subgroup OR 1.38 (1.19–1.60; I²=25%)	Mostly cross-sectional; heterogeneity; only 3 cohorts
C. Mendelian randomization						
Tan 2024 (14)	Bidirectional two-sample MR (European GWAS); CP 3,046/195,395; NAFLD 894/217,898	GWAS-defined CP; SNP instruments	Genetically predicted NAFLD	MR; heterogeneity and pleiotropy tested	CP→NAFLD: IVW β=0.132, p=0.006; reverse direction NS (p=0.081)	European ancestry; few NAFLD cases; limited instrument strength
Cheng 2024 (15)	Bidirectional two-sample MR; discovery + FinnGen validation	GWAS periodontitis; 5/18 SNP instruments	Genetically predicted NAFLD	Sensitivity analyses; smoking/T2DM-related SNPs excluded	No evidence either direction: PD→NAFLD OR 1.04 and 1.07; reverse direction NS	Few/relaxed-threshold instruments; GWAS phenotype differences; limited power
Qiao 2024 (16)	Bidirectional MR; European ancestry; PD n=34,615; liver GWAS n≈334,000–342,000	GWAS-defined periodontitis; SNP instruments	NAFLD, NASH, fibrosis and cirrhosis	Sensitivity analyses; no major heterogeneity/pleiotropy	NAFLD→PD OR 1.094 (1.006–1.189); cirrhosis→PD OR 1.138 (1.001–1.294); PD→liver outcomes NS	Small/borderline effects; multiple outcomes; European ancestry/limited PD instrument power
Zhao 2026 (101)	Systematic review of 78 MR studies; 34 meta-analyzed	GWAS-derived periodontitis instruments	NAFLD among systemic outcomes	Pooled IVW/MR-Egger; sensitivity/publication-bias analyses	IVW PD→NAFLD OR 1.10 (1.04–1.17); MR-Egger OR 1.09 (0.99–1.22), NS	Weak PD instruments; mainly European ancestry; possible sample overlap/method dependence
D. Interventional evidence						
Kamata 2022 (29)	Multicenter open-label RCT, Japan; n=40; SRP vs tooth-brushing control; 12-w primary, 60-w follow-up	Moderate periodontitis by PPD/BOP; SRP intervention	Primary: ALT; secondary: serum P. gingivalis IgG; exploratory MRI-PDFF/MRE/VCTE-CAP	Randomization; no meaningful short-term weight/BMI change	12 w: ALT −12 vs +1 U/L (difference −12, 95% CI −20 to −5); P. gingivalis IgG also decreased. Imaging improved short-term; 60-w MRI-PDFF −3.3% vs +0.17%, but durability varied across measures	Very small sample; open-label; imaging exploratory/secondary and long-term persistence inconsistent

### 1.3 From microbial signals to myeloid immune reprogramming

Beyond epidemiological associations, attention has shifted to the biological mechanisms that could link periodontitis to MASLD. Existing frameworks center largely on the oral–gut–liver axis: oral dysbiosis, periodontal pathogens, and their products may spread beyond the oral cavity, alter intestinal microbial ecology and barrier function, and ultimately change the microbial signals reaching the liver through the portal circulation (10, 30, 31). In experimental models, oral administration of *Porphyromonas gingivalis* alters gut microbial composition and aggravates steatotic liver injury, and extracellular vesicles derived from periodontal pathogens can promote hepatic steatosis through Toll-like receptor 2 (TLR2)-dependent signaling (32–34). These microbial signals also engage inflammatory sensing and amplification pathways—including lipopolysaccharide (LPS)–TLR signaling, nuclear factor kappa B (NF-κB) activation, and NLRP3 inflammasome pathways—that have been implicated in chronic liver inflammation and metabolic liver injury (35–37). Together, these mechanisms explain mainly how periodontal danger signals reach distant tissues and are sensed and amplified within the liver. A recent review proposed a “liver–gut–immune–oral axis” in which trained immunity acts as a systemic bridge linking chronic liver disease and periodontitis (38). In this review, we focus on a more downstream and mechanistically defined question: whether the myeloid output generated under conditions of periodontitis can interact with the stage-dependent immune microenvironment of the MASLD liver. Specifically, we address three interrelated issues. First, we examine the functional state of circulating neutrophils and monocytes before their entry into the liver, as this represents the downstream consequence of hematopoietic stem and progenitor cell (HSPC) reprogramming and myeloid preconditioning. Second, we consider how the capacity of the liver to recruit, retain, and reshape these cells changes across distinct stages of MASLD, including steatosis, MASH, and fibrosis. Third, we critically evaluate the currently available evidence linking these processes across organs. Because direct evidence for this cross-organ connection remains lacking, we explicitly define this gap as a missing “bridging” link and, on this basis, propose a set of experimentally testable hypotheses to determine whether and how periodontitis-associated myeloid reprogramming contributes to stage-specific immune remodeling in MASLD.

Microbial signal transmission alone, however, may not account for the persistence of the systemic effects associated with chronic periodontitis. A key unresolved question is whether these effects depend solely on the continuous influx of microbial products and soluble inflammatory mediators, or whether chronic periodontal inflammation can also reset the basal responsiveness of the host innate immune system. Studies of trained immunity show that inflammatory or microbial stimuli can induce durable metabolic, transcriptional, and epigenetic reprogramming of hematopoietic stem and progenitor cells (HSPCs), thereby shaping subsequent myelopoiesis and the functional state of their myeloid progeny (39–43). Experimental studies have further shown that periodontitis can induce a comparable form of maladaptive innate immune training within the bone marrow (44). We therefore regard microbial products and soluble inflammatory mediators not as the sole mediators of the periodontitis–MASLD connection but as potential upstream triggers of longer-lasting reprogramming of systemic myeloid immunity.

A study by Li et al. provides the most direct evidence to date. Experimental periodontitis in mice induced epigenetic reprogramming of bone marrow HSPCs and sustained enhancement of myelopoiesis, generating myeloid progeny with increased inflammatory preparedness; this trained phenotype persisted after resolution of local periodontal inflammation. When bone marrow from previously exposed mice was transplanted into periodontitis-naive recipients, subsequently induced inflammatory arthritis was more severe and was accompanied by increased recruitment of monocytes and neutrophils to inflamed joints (44). These results are consistent with classic studies showing that long-lived innate immune memory can be encoded at the level of HSPCs, sustaining altered myeloid output well beyond the original inflammatory stimulus (39–41). The systemic consequences of periodontitis may therefore depend not only on the continued presence of circulating microbial products or inflammatory mediators but also on persistent reprogramming of the bone marrow compartment. Meanwhile, the focus of MASLD immunology has moved from a general concept of “hepatic inflammation” toward the heterogeneity, ontogeny, fate, and tissue niches of individual myeloid populations (45, 46); as the disease progresses from simple steatosis to MASH and fibrosis, the hepatic myeloid landscape is continually remodeled rather than fixed in composition (47–50).

Much of the MASLD literature, however, has focused on how myeloid cells are shaped after they enter the hepatic microenvironment (47, 48). Less attention has been paid to whether circulating myeloid cells already carry an inflammatory program established by chronic inflammatory conditions outside the liver. This distinction matters particularly for periodontitis. Experimental periodontitis can induce sustained HSPC reprogramming and alter the inflammatory preparedness of subsequently generated monocytes and neutrophils (39, 41, 44); in parallel, recruited monocytes, monocyte-derived macrophages, and neutrophils contribute to hepatic immune remodeling during steatosis, steatohepatitis, and fibrosis (47, 48, 50). Whether periodontitis-induced myeloid memory intersects with hepatic myeloid remodeling during MASLD progression has not been established.

This defines the central knowledge gap. If circulating myeloid cells recruited into the MASLD liver carry an inflammatory imprint established by periodontitis-associated systemic inflammation, their subsequent differentiation, inflammatory responsiveness, and tissue-remodeling functions may differ from those of cells generated under homeostatic conditions. Direct experimental evidence that periodontitis-induced HSPC or myeloid reprogramming modifies the hepatic immune landscape of MASLD is still lacking, so this possibility should be regarded as a mechanistically plausible working hypothesis rather than an established causal pathway.

On these grounds, we use the “periodontium–bone marrow–circulating myeloid cell–liver” axis as a heuristic working framework to organize the currently available evidence, rather than as an established causal pathway. Chronic periodontal inflammation may persistently influence bone marrow HSPCs and alter the basal inflammatory program of newly generated monocytes and neutrophils (39, 40, 44, 51), whereas the MASLD liver undergoes disease-stage-specific changes in its capacity to recruit and reshape peripheral myeloid cells (47–49). The key unresolved question is therefore whether myeloid cells generated under conditions of periodontitis acquire functional alterations before hepatic recruitment and, if so, whether these altered cells are subsequently recruited to and further reshaped within the immune microenvironment of the MASLD liver in a disease-stage-dependent manner. This framework is summarized in **Figure 1**.

**Figure 1:**
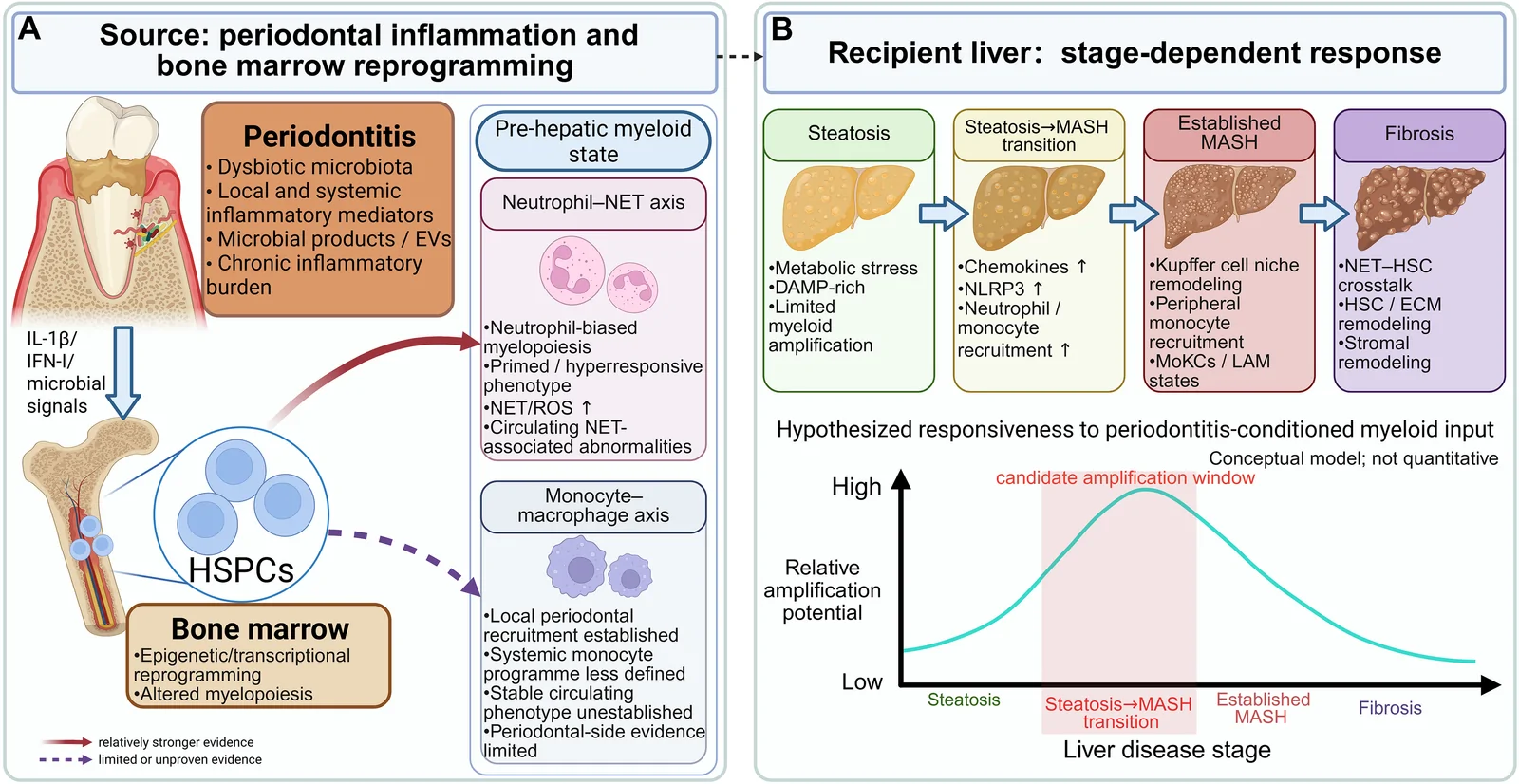
Proposed framework linking periodontitis-conditioned myeloid states to stage-dependent hepatic responses in MASLD. **(A)** Chronic periodontal inflammation provides persistent microbial and inflammatory signals that can reprogram bone marrow hematopoietic stem and progenitor cells (HSPCs) and alter subsequent myeloid output. Evidence is stronger for neutrophil-biased myelopoiesis and a primed, hyperresponsive neutrophil phenotype, whereas stable periodontitis-specific systemic reprogramming of monocytes remains less well defined. **(B)** The MASLD liver is conceptualized as a stage-dependent recipient niche. Simple steatosis provides a metabolically stressed but relatively limited inflammatory environment; the steatosis-to-MASH transition is accompanied by increasing chemotactic signaling, innate immune activation, and peripheral myeloid recruitment; established MASH involves Kupffer cell niche remodeling and increased contribution of monocyte-derived macrophages; and fibrosis increasingly engages NET–hepatic stellate cell interactions and stromal remodeling. The dashed connection between **(A, B)** represents the central unresolved step: whether periodontitis-conditioned myeloid cells enter the MASLD liver, retain functionally relevant features of their pre-hepatic state, and modify hepatic inflammation or fibrosis. The “candidate amplification window” refers to a hypothesized period during the steatosis-to-MASH transition when enhanced inflammatory signaling and increased myeloid recruitment may confer relatively greater hepatic responsiveness to periodontitis-conditioned myeloid input. The y-axis represents the relative amplification potential of the hepatic response to this specific input, reflecting a hypothesized additional response rather than the overall level of liver inflammation. The proposed increase in hepatic responsiveness around the steatosis-to-MASH transition is conceptual and is not intended to represent a quantitative relationship, a fixed biological time interval, or a directly measured amplification effect. Created in BioRender. xudong, S. (2026) https://BioRender.com/98pvyr0.

## 2 The periodontal origin of systemic inflammatory signals

### 2.1 Periodontal myeloid niche: a source of persistent inflammatory signals

Integrated single-cell transcriptomic and spatial analyses have demonstrated that periodontitis is characterized by a spatially compartmentalized inflammatory immune niche rather than a diffuse inflammatory infiltrate (52). Easter et al. integrated four single-cell RNA sequencing datasets comprising 34 samples and 105,918 cells and showed that lesional keratinocytes markedly upregulated multiple chemotactic and inflammatory mediators, including CXCL1, CXCL3, CXCL8, CCL20, IL1A, and IL1B (52). Spatial analyses further revealed a distinct immune architecture, in which the junctional epithelium was predominantly enriched in myeloperoxidase (MPO)-positive neutrophils and CD14^+^/CD68^+^ monocytes/macrophages, whereas the sulcular epithelium preferentially accumulated adaptive immune cells, including T cells, B cells, and even tertiary lymphoid structures (52). Collectively, this spatially organized inflammatory niche provides a structural basis for persistent local recruitment and maintenance of myeloid inflammation within the periodontium (52, 53).

Under physiological conditions, periodontal homeostasis depends on continuous neutrophil production in the bone marrow, migration of these cells through the circulation to the gingival crevice, and frontline surveillance of the dental biofilm (53, 54). Disruption of periodontal homeostasis is therefore characterized not simply by increased neutrophil numbers but by persistent recruitment together with dysregulated effector programs, including excessive activation, reactive oxygen species (ROS) production, degranulation, and the formation of neutrophil extracellular traps (NETs). These alterations ultimately shift physiological barrier defense toward self-amplifying myeloid inflammation (53, 55, 56). Periodontitis therefore establishes not merely an increased local inflammatory burden but an immune niche that continuously generates myeloid inflammatory signals and serves as a potential source of subsequent systemic immune remodeling.

### 2.2 Microbial products and soluble inflammatory mediators: triggers rather than primary inflammatory relays

Periodontal microbial products can breach the local tissue barrier and exert biological effects in distant organs (30, 32–34). Kim et al. showed that EVs derived from the periodontal pathogens *Filifactor alocis* and *Porphyromonas gingivalis* induced hepatic steatosis in mice through activation of the TLR2–plasminogen activator inhibitor-1 (PAI-1) signaling pathway; serum TLR2-activating activity was also positively associated with circulating PAI-1 levels in humans, supporting the potential clinical relevance of this mechanism (33). Consistent with these findings, pathogen-derived EVs and host-cell exosomes generated within the periodontal inflammatory milieu have been proposed as mediators capable of escaping the periodontal lesion, entering the circulation, and influencing distant organs (33, 57).

More direct evidence comes from a study showing that *P. gingivalis*-derived outer membrane vesicles (OMVs) carrying peptidylarginine deiminase (PPAD) preferentially accumulated in the liver under experimental conditions and disrupted hepatic lipid metabolism through the NPAS2/CYP4A10 pathway. In obese mice, periodontitis further aggravated the MASLD phenotype, indicating that periodontal OMVs can promote metabolic dysfunction beyond the oral cavity (58). Gingipains have likewise been shown to cleave the insulin receptor directly through their proteolytic activity, providing further evidence that the biological effects of periodontal virulence factors are not restricted to periodontal tissues (59). Together, these findings show that periodontal microbial products can cross the local tissue barrier, enter the systemic circulation, and exert biological effects in distant organs (33, 58).

Direct microbial dissemination alone, however, cannot explain why periodontitis may produce long-lasting systemic effects that resemble host immune memory. The more important question is whether these microbial stimuli reprogram the host myeloid immune system and thereby establish a sustained inflammatory state. Beyond microbial products, periodontitis may promote systemic inflammatory activation through soluble inflammatory mediators, including C-reactive protein (CRP), interleukin-6 (IL-6), IL-1β, and tumor necrosis factor alpha (TNF-α) (60–62). These mediators are more likely to act as upstream triggers that condition the bone marrow inflammatory environment than as direct vectors sustaining long-term inflammatory memory.

Disseminated microbial products and soluble inflammatory mediators therefore act primarily as triggers and environmental conditioning signals. The key unresolved question is how these upstream signals remodel the host myeloid immune system to establish sustained systemic inflammatory propagation and amplification (44, 51).

### 2.3 Bone marrow reprogramming: the strongest systemic evidence from periodontitis

Circulating neutrophils, whose lifespan ranges from hours to several days depending on the experimental approach, and monocytes are short-lived, so mature myeloid cells alone are unlikely to account for long-lasting systemic innate immune memory (51, 63). If periodontitis induced only transient phenotypic changes in existing circulating cells, the systemic inflammatory response would be expected to fade as these cells turn over. The persistence of altered myeloid responsiveness across successive generations of short-lived effector cells therefore points to upstream reprogramming of hematopoiesis, particularly at the level of HSPCs, allowing newly generated myeloid cells to inherit a durable proinflammatory functional program (39, 40, 44, 51).

Experimental studies provide direct evidence that periodontitis can induce maladaptive innate immune training within the bone marrow. Periodontal inflammation causes epigenetic and transcriptional reprogramming of HSPCs and the sustained generation of inflammatory myeloid progeny. The trained phenotype is transmissible by bone marrow transplantation, demonstrating that hematopoietic reprogramming—rather than persistence of mature circulating cells—underlies the systemic inflammatory memory induced by periodontitis. Mechanistically, interleukin-1 (IL-1) signaling within HSPCs is required to establish this maladaptive training program and contributes to enhanced inflammatory susceptibility in recipient animals (44).

Within the broader framework of hematopoietic reprogramming, experimental periodontitis in a Dnmt3a R878H-driven clonal hematopoiesis model influenced the competitive fitness, lineage bias, and myeloid output of hematopoietic stem cells. Together with evidence from HSPC profiling studies, these results support the view that periodontal inflammation remodels the hematopoietic compartment rather than merely activating peripheral immune cells (44, 64, 65).

A 2025 Theranostics study integrating single-cell transcriptomic and chromatin-accessibility analyses delineated the lineage features of periodontitis-induced HSPC reprogramming. Periodontitis shifted HSPCs toward neutrophil-lineage differentiation and induced sustained epigenetic changes, and type I interferon (IFN-I) signaling was implicated as a key process linking periodontal inflammatory stimuli to HSPC reprogramming and altered myeloid output (65). Resolution of periodontal inflammation distinguished quantitative from functional recovery: after ligature removal, neutrophil numbers and the HSPC neutropoietic bias largely returned to baseline, whereas neutrophil hyperresponsiveness and some residual inflammatory susceptibility persisted, indicating that periodontitis-induced immune training is durable and only partially reversible (44, 65). However, the duration of this residual trained state after resolution of periodontal inflammation remains unclear, particularly in humans, where longitudinal assessment of hematopoietic and myeloid changes after periodontal treatment is lacking. We therefore identify the persistence of this imprint after periodontal resolution as an open question for future investigation.

These experimental observations align with recent conceptual advances in trained immunity. Zhao et al. proposed that systemic mediators generated during periodontitis—particularly IL-1β and type I interferons—reprogram bone marrow HSPCs to promote sustained myelopoiesis and the production of hyperresponsive neutrophils (66); Hajishengallis, Netea, and Chavakis likewise argued that long-lasting trained immunity in chronic inflammatory diseases depends on metabolic, transcriptional, and epigenetic adaptations within bone marrow progenitor compartments rather than on mature innate immune cells alone (39, 67). Whether the periodontitis-induced HSPC–neutropoiesis axis contributes directly to MASLD remains undetermined; current experimental evidence that periodontitis aggravates MASLD points mainly to other mechanisms (58, 68).

### 2.4 Neutrophil priming and aberrant NET formation

Once HSPC reprogramming has established a persistent state of myeloid susceptibility, neutrophils may emerge as a key functional output. In experimental periodontitis, NET formation precedes overt Th17 accumulation, and degradation of NETs or inhibition of NET-associated components markedly attenuates subsequent IL-17/Th17 responses and alveolar bone destruction (69). NETs are therefore not merely downstream products of inflammation but actively initiate inflammatory amplification cascades in periodontal disease.

NETs may also act as immunomodulatory signals that promote local Th17 responses; IL-17 in turn enhances neutrophil recruitment and activation, forming a self-amplifying feed-forward loop that sustains periodontal inflammation (55, 69). In patients with severe periodontitis, NET-associated signatures are elevated not only within periodontal lesions but also in the peripheral circulation: NET-related complexes and extracellular histones carrying specific post-translational modifications are detectable in blood and correlate with disease severity (69). Dysregulated neutrophil activation is thus not confined to the local periodontal lesion but may also generate a detectable systemic inflammatory phenotype. Circulating NET-associated complexes and aberrantly modified extracellular histones in patients with severe periodontitis constitute important human evidence linking local periodontal inflammation to systemic immune activation (69).

Combined with the HSPC reprogramming studies discussed above, these findings suggest that periodontitis alters both the neutrophil production program and the functional state of these cells, yielding a persistently primed phenotype characterized by enhanced ROS production, increased inflammatory cytokine secretion, and increased NET formation. Continued exposure to the periodontal inflammatory microenvironment may further maintain and amplify this hyperresponsive state, thereby promoting further NET formation and inflammatory signal amplification (55, 56, 65).

### 2.5 Monocytes: established local recruitment, undefined systemic reprogramming

Compared with neutrophils, monocyte abnormalities in periodontitis manifest mainly as continuous recruitment into inflamed tissues (52, 70). Persistent inflammation drives the accumulation of CD14^+^/CD68^+^ cells, indicating that the myeloid composition of periodontal lesions depends on continuous replenishment from the circulation rather than on expansion of resident macrophages alone (52, 70, 71). Recent single-cell studies indicate that local regulatory pathways such as the IL-33/ST2 axis shape periodontal macrophage states, underscoring the dominant role of the local tissue niche in determining myeloid states (71).

Persistent local recruitment, however, does not equate to systemic expansion of inflammatory monocytes. Current evidence is insufficient to show that periodontitis increases the proportion of circulating inflammatory monocytes or induces stable systemic reprogramming of specific monocyte subsets. Although periodontitis can induce HSPC remodeling, the available evidence supports preferential skewing toward the neutrophil lineage rather than sustained inflammatory monocyte expansion (44, 65). Myeloid adaptation at the bone marrow level is thus relatively well supported, whereas durable systemic reprogramming of monocytes remains unestablished.

## 3 The MASLD liver as a stage-dependent recipient of myeloid input

This section conceptualizes the MASLD liver as a dynamic recipient niche that may shape how incoming myeloid cells are recruited, retained, and functionally reprogrammed. As MASLD progresses from isolated steatosis (MASL) through the steatosis-to-MASH transition, established MASH, and fibrosis, the hepatic immune landscape is progressively remodeled, creating stage-specific conditions that may differentially shape the impact of peripheral myeloid inputs (**Figure 1B**).

### 3.1 The sensitized steatotic liver: primed but not yet permissive

Simple steatosis does not leave the liver immunologically quiescent. Nutrient excess, adipose tissue dysfunction, and increased delivery of free fatty acids impose a persistent metabolic burden on the liver, producing hepatocellular lipotoxicity, oxidative stress, mitochondrial and endoplasmic reticulum stress, and the release of damage-associated molecular patterns (DAMPs). The steatotic liver thus presents a DAMP-rich background of innate immune priming (72–74).

These DAMPs and other injury-associated signals can be sensed by Kupffer cells (KCs) and other innate immune cells, initiating or amplifying inflammation through TLR pathways and, as steatosis progresses toward steatohepatitis, the NLRP3 inflammasome (74–76); evidence for TLR9- and NLRP3-dependent amplification derives mainly from NASH/MASH models rather than from simple steatosis. At this stage, the liver has already departed from normal immunometabolic homeostasis.

Here, we use the term “sensitized liver” to describe a steatotic liver exposed to persistent metabolic stress but not yet showing the fully inflammatory phenotype of MASH. The term is a working concept rather than a new pathologic classification, and it emphasizes that such a liver may have a lower threshold for responding to additional inflammatory stimuli.

At the stage of simple steatosis, the liver therefore already has the molecular machinery to receive exogenous inflammatory signals but has not yet developed an inflammatory niche capable of sustained recruitment and amplification of myeloid input. This may help explain why the impact of systemic inflammatory stimuli on MASLD progression is markedly stage-dependent.

### 3.2 The steatosis-to-MASH transition: a candidate window for heightened immune amplification

During the transition from steatosis to MASH, metabolic injury persists while innate immune amplification and peripheral myeloid cell recruitment become progressively more prominent. Lipotoxicity, oxidative stress, injured hepatocytes, and the DAMPs they release provide sustained endogenous stimuli that activate KCs and inflammatory circuits involving the NLRP3 inflammasome, IL-1β, TNF-α, and chemokines (76, 77). Neutrophils and monocyte-derived cells are increasingly recruited to the liver and participate in local inflammation (78, 79). The liver at this stage is thus exposed both to persistent endogenous injury signals and to increasing peripheral myeloid input, potentially increasing its capacity to convert a pre-existing systemic myeloid hyperresponsive state into local inflammatory amplification. Metabolic stress establishes the substrate for disease progression, whereas amplification of innate immunity may help drive the liver across the threshold from simple steatosis to inflammatory MASH.

Consistent with this model, Zhou et al. showed that lipid droplets released from injured steatotic hepatocytes promote the induction of TREM2^+^ MASH-associated macrophages and drive MASH through MS4A7-dependent NLRP3 inflammasome activation (77). The hepatic microenvironment also becomes increasingly permissive to neutrophil recruitment: CXCL1 and IL-8 are markedly increased in human NASH compared with simple fatty liver, and experimentally enhancing these neutrophil-recruiting signals can promote the transition from steatosis to NASH (79). Guillot et al., combining transcriptomic profiling and imaging of human liver tissue with mechanistic mouse studies, further identified the accumulation of monocyte-derived macrophages as a prominent stage-associated immune alteration during NASH progression (78). The steatosis-to-MASH transition thus represents not only a continuation of metabolic injury but also a change in the liver’s capacity to recruit and respond to peripheral myeloid input.

As the liver approaches MASH, persistent metabolic injury is accompanied by stronger chemotactic signaling, inflammasome activation, and recruitment of peripheral myeloid cells, creating a microenvironment increasingly capable of receiving and amplifying systemic inflammatory myeloid input (77–79). This is consistent with the finding that periodontitis can induce sustained reprogramming of myelopoiesis and the generation of primed neutrophils (44, 65).

### 3.3 MASH: disruption of the Kupffer cell niche and increasing contribution of peripheral myeloid cells

Once MASH is established, remodeling of the hepatic myeloid compartment involves not merely further escalation of inflammation but a substantial change in macrophage ontogeny and niche organization. Under homeostatic conditions, resident KCs stably occupy the sinusoidal macrophage niche; in MASH, they undergo loss of identity, functional impairment, reduced self-renewal, and cell death, while recruitment of circulating monocytes increases. Recruited cells give rise to heterogeneous monocyte-derived macrophage states, including monocyte-derived KCs (MoKCs), lipid-associated macrophages (LAMs), and other disease-associated populations (47, 80). The relative contribution of peripheral myeloid cells to the hepatic macrophage compartment therefore increases with disease progression. In the human liver, single-cell analyses identify both resident KCs and monocyte-derived macrophage populations, with CD14^+^ monocyte/macrophage abundance correlating with the degree of steatosis (48); NASH-associated injury is further accompanied by erosion of canonical KC signatures and the emergence of TREM2- and CD9-associated macrophage programs (47).

Zhang et al. provided further mechanistic evidence for disruption of the KC niche. In MASH, NCF1-dependent ROS produced by macrophages promote the formation of oxidized phospholipids, which induce TLR4-dependent hepcidin production in hepatocytes. Increased hepcidin then promotes iron accumulation and ferroptosis in KCs, accompanied by increased infiltration of monocyte-derived macrophages (81). KC loss and niche remodeling can therefore increase the contribution of recruited monocyte-derived cells to the hepatic macrophage pool, although KC death is not an absolute prerequisite for monocyte infiltration.

Beyond niche opening, chemotactic signals further determine how peripheral monocytes enter the inflamed liver. Damaged hepatocytes, activated macrophages, and other hepatic cells can produce chemokines including CCL2, thereby promoting recruitment of CCR2^+^ circulating monocytes to the chronically inflamed liver (82). Once recruited, these cells can differentiate into distinct monocyte-derived macrophage states according to local environmental cues. Within the present framework, the CCL2–CCR2 axis is therefore particularly useful for explaining how peripheral monocytes are recruited into the inflamed liver, while recognizing that this pathway can itself contribute to MASH-associated inflammation.

Recruited myeloid cells are also reshaped after entering the liver. Spatial and single-cell studies show that hepatic macrophage identities are strongly influenced by the local microenvironment: lipid exposure promotes LAM programs, whereas distinct cellular interactions support KC identity and other macrophage states (47, 83). Spatial genomics analyses likewise indicate that immune cells in MASLD are not uniformly distributed throughout the liver but are organized within spatially distinct regions of steatosis, injury, and fibrosis (84). Periodontitis may thus alter the functional starting state of circulating myeloid cells, whereas their subsequent differentiation, inflammatory activity, and reparative programs are likely to be strongly shaped by the hepatic niche they encounter.

### 3.4 MASH-to-fibrosis transition: context-dependent TREM2^+^ macrophage programs and the NET–HSC experimental bridge

TREM2 should not be interpreted simply as a unidirectional profibrotic macrophage marker. During MASH progression, TREM2^+^ disease-associated macrophage programs emerge at sites of hepatocellular injury. Lipid droplets released from injured steatotic hepatocytes can induce TREM2^+^ MASH-associated macrophages, within which MS4A7 promotes NLRP3-dependent inflammatory amplification (77). These findings, however, do not establish TREM2 itself as a proinflammatory driver.

Conversely, TREM2^+^ macrophages can acquire prominent reparative functions during disease improvement. After vertical sleeve gastrectomy, TREM2^+^ LAMs show increased lysosomal activity, reduced inflammatory activation, and enhanced efferocytosis of lipid-laden apoptotic hepatocytes, and TREM2 deficiency impairs these beneficial effects (85). During experimental MASH regression, TREM2^+^ LAMs are similarly enriched in disease-resolving programs and contribute to collagen degradation and fibrosis resolution (80). TREM2^+^ macrophages should therefore be viewed as a disease-associated macrophage program whose functional consequences depend strongly on disease stage and the local microenvironment, rather than as a uniformly profibrotic population.

Unlike the context-dependent effects of TREM2^+^ macrophages, NETs provide a more direct experimental link between myeloid inflammation and the fibrotic program. Xia et al. showed that NET formation increases during MASH fibrosis, whereas genetic or pharmacological reduction of NET formation attenuates experimental fibrosis. Mechanistically, NETs induce metabolic reprogramming of hepatic stellate cells (HSCs) and promote their activation, proliferation, and migration, thereby directly linking neutrophil effector activity to fibrogenesis (86). This study offers one of the clearest experimental bridges between myeloid inflammation and HSC-dependent fibrosis.

Babuta et al. further showed that when repeated alcohol exposure is superimposed on MASH, NETs activate both HSCs and monocytes through NLRP3-dependent sensing, thereby establishing an additional amplification loop that links neutrophilic inflammation to accelerated fibrosis (87).

As inflammatory MASH progresses toward fibrosis, the hepatic response to peripheral myeloid input thus extends beyond inflammatory amplification and increasingly intersects with stromal remodeling. Disruption of the Kupffer cell niche increases the contribution of recruited monocyte-derived macrophages to hepatic immune remodeling, while neutrophils and NET-associated signals can directly promote HSC activation and fibrogenic responses (47, 86).

## 4 Evidence appraisal: two supported ends, a missing bridge

Mechanistic plausibility cannot substitute for causal evidence in humans; the aim here is to propose and examine an unproven but biologically plausible model rather than to assert causality. The evidence is distributed asymmetrically across the two candidate axes: relatively direct evidence exists at each end—the periodontal end and the hepatic end—whereas the connecting step, namely, whether periodontitis-conditioned myeloid cells actually enter the MASLD liver and modify its immune remodeling, has not been demonstrated. No human study has prospectively demonstrated the complete sequence from periodontal inflammatory burden to systemic myeloid alteration to hepatic immune remodeling to MASLD progression. Population studies generally support clinical co-occurrence, longitudinal studies suggest a possible temporal association, and preliminary interventional studies suggest that reducing periodontal inflammatory burden may improve selected hepatic surrogate endpoints, but none of these studies has serially assessed systemic myeloid mediators (11, 12, 22, 29, 88). The following sections appraise the evidence by level.

### 4.1 The neutrophil–NET axis: evidence at both ends, a gap in between

Neutrophil dysregulation and aberrant NET formation are supported by relatively direct human and experimental evidence in periodontitis. Periodontal lesions are characterized by persistent neutrophil recruitment and NET formation, and a NET-related neutrophil population has been identified in human periodontitis tissues (89). Patients with severe periodontitis also exhibit increased NET complexes and extracellular histones bearing NET-associated post-translational modifications, not only within periodontal lesions but also in peripheral blood, indicating that these abnormalities are not confined to the local periodontal lesion (69).

Experimental periodontitis can further reprogram HSPCs toward neutrophil-biased differentiation and generate circulating neutrophils with a primed, hyperresponsive phenotype (65). Neutrophil abnormalities in periodontitis may therefore extend beyond increased local accumulation and NET formation to involve broader alterations in the systemic myeloid state. Evidence for sustained bone marrow reprogramming, however, derives predominantly from experimental models, and its persistence and biological significance in human periodontitis remain to be established.

In MASLD, neutrophils and NETs have likewise moved from being viewed as accompanying inflammatory markers to being recognized as functionally relevant participants in disease progression. Neutrophil infiltration and NET formation increase during MASH-associated fibrosis, whereas depletion of NETs with DNase I or genetic inhibition of NET formation attenuates fibrosis in experimental models (86). Mechanistically, NETs induce metabolic reprogramming of hepatic stellate cells (HSCs) and promote their activation, proliferation, and migration, thereby translating neutrophil effector activity into a fibrogenic response. When recurrent alcohol exposure is superimposed on MASH, NETs also activate both HSCs and monocytes through NLRP3-dependent sensing, further reinforcing the link between myeloid inflammation and tissue remodeling (87). On the recipient side, the MASH liver thus provides a pathologic environment capable of responding to and amplifying neutrophil- and NET-associated signals.

The critical evidence gap lies between these two ends of the proposed axis. No study has yet shown that a periodontitis-conditioned neutrophil population traffics to the MASLD liver and drives MASH or fibrosis, nor has circulating periodontal-associated NET burden been shown to reach the liver and causally contribute to hepatic disease progression. Systemic neutrophil/NET abnormalities in periodontitis and pathogenic NET activity in MASH are thus each supported independently, but the cross-organ relay connecting them remains based on biological plausibility rather than direct cell-tracing or causal intervention evidence.

The neutrophil–NET axis can therefore be regarded as one of the most coherent mechanistic hypotheses linking periodontitis to MASLD, but not as an established causal pathway. A more plausible model is not that “periodontal NETs directly cause MASH,” but that chronic periodontitis establishes a more reactive systemic neutrophil state and increases NET-associated inflammatory pressure. When such altered myeloid input encounters a MASLD liver already permissive to neutrophil recruitment and NET-mediated inflammatory and fibrogenic amplification, it may further amplify local inflammation and fibrosis. The central unresolved question is thus not whether NET abnormalities occur in both diseases, but whether periodontitis alters the initial functional state of neutrophils entering the MASLD liver and whether this preconditioning ultimately changes the trajectory of liver disease.

### 4.2 The monocyte–macrophage axis: strong hepatic evidence, weak periodontal support

The monocyte–macrophage axis shows a more pronounced asymmetry in the strength of evidence between periodontitis and MASLD. Monocytes are recruited to inflamed periodontal tissues, a process in which local CCL2 signaling plays a central role in diabetes-associated periodontitis (90). Experimental studies further indicate that chronic periodontal inflammation can influence upstream myelopoiesis and increase the inflammatory preparedness of myeloid progeny (44). Human studies have also reported modest alterations in circulating monocyte subsets in periodontitis. However, there is currently insufficient evidence to define a stable, periodontitis-specific circulating monocyte program with a clearly established recruitment phenotype, such as persistent expansion of CCR2^+^ inflammatory monocytes. Thus, the periodontal evidence currently more strongly supports a broader state of systemic myeloid activation than a specific circulating monocyte program.

By contrast, monocyte recruitment and macrophage remodeling are well established in MASLD. During progression to MASH, chemotactic pathways, including the CCL2–CCR2 axis, promote recruitment of circulating monocytes into the liver (82). Human tissue studies further show that accumulation of bone marrow-derived monocytic macrophages increases with disease progression and represents a prominent feature of the hepatic immune landscape in NASH/MASH (78). Once recruited, monocytes are exposed to lipid overload, tissue injury, and fibrotic niche signals and can differentiate into heterogeneous macrophage states, including monocyte-derived macrophages, monocyte-derived KCs (MoKCs), and LAMs (47, 80). Single-cell and spatial studies further demonstrate that these macrophage states are strongly shaped by the local hepatic microenvironment and exhibit substantial spatial and temporal heterogeneity (83).

Importantly, myeloid remodeling in MASH is not restricted to the liver. Circulating monocytes from patients with MASH display a distinct immunometabolic phenotype characterized by increased glycolysis and mitochondrial respiration, enhanced complex II activity, and increased ROS-linked inflammatory output (91). These findings indicate that the MASLD host itself can exhibit systemic monocyte reprogramming in addition to local hepatic macrophage remodeling.

The central gap in this axis is therefore not whether the MASH liver can recruit circulating monocytes, but whether the recruited cells were previously conditioned by periodontitis. To date, there is no lineage-tracing evidence demonstrating that periodontitis-conditioned circulating monocytes preferentially enter the MASLD liver or subsequently adopt a specific hepatic macrophage fate. Even if periodontitis alters the inflammatory responsiveness of circulating monocytes, their fate after entering the liver is likely to be reshaped by local metabolic stress, tissue injury, and spatial niche signals (47, 83). Periodontitis-associated systemic monocyte activation therefore cannot be directly equated with the generation of any particular intrahepatic macrophage subset.

At present, the monocyte–macrophage axis has a strong mechanistic foundation on the MASLD side, encompassing monocyte recruitment, niche-dependent differentiation, and tissue remodeling. By contrast, whether periodontitis induces a stable, traceable, and functionally relevant systemic monocyte program remains insufficiently defined. The cross-organ connection is therefore biologically plausible, but the direct evidence for this connection remains weaker than that for the neutrophil–NET axis.

### 4.3 Resolution failure: a shared state of inflammatory susceptibility

Beyond the two cross-organ myeloid axes discussed above, periodontitis and MASLD may share another inflammatory background: an impaired capacity to resolve inflammation. Effective resolution normally requires clearance of apoptotic neutrophils by macrophages, functional reprogramming of macrophages toward pro-resolving states, and coordinated activation of specialized pro-resolving pathways. In periodontitis, defective efferocytosis and impaired pro-resolving programs can prolong neutrophil-dominated inflammation and delay restoration of periodontal homeostasis (92, 93). Recent human single-cell analyses identified a resolution-deficient immune landscape in periodontitis, characterized by impaired macrophage efferocytosis and reduced resolvin D1 biosynthetic capacity (93).

A similar defect is evident in MASH. Prolonged metabolic stress can impair macrophage clearance of dying hepatocytes by disrupting TREM2-dependent efferocytosis, promoting accumulation of dead cells and sustaining inflammatory signaling (94). Conversely, during MASH regression, TREM2^+^ macrophages acquire enhanced phagocytic, lipid-handling, and matrix-remodeling programs that contribute to restoration of tissue homeostasis and fibrosis resolution (80). Failure to clear damaged cells efficiently and to transition toward reparative macrophage states may therefore contribute to persistent inflammation in both periodontal and hepatic tissues.

Such a shared resolution-deficient state could increase the susceptibility of both organ systems to persistent inflammatory input. Periodontal tissues may be less able to terminate local myeloid inflammation, whereas a metabolically stressed liver with defective resolution machinery may be less able to buffer additional inflammatory signals arriving from the circulation. Unlike direct neutrophil or monocyte trafficking, this model does not require a specific cell or mediator to travel from the periodontal lesion to the liver; rather, it is best viewed as a shared background of impaired inflammatory control that may increase vulnerability to sustained myeloid stimulation.

Whether this state is reversible remains an important open question. Experimental resolution of periodontitis can reverse HSPC neutropoietic bias and normalize circulating neutrophil numbers; however, neutrophil hyperresponsiveness—including enhanced ROS production, inflammatory mediator release, and NET formation—can remain partially elevated during the recovery period (65). Human data similarly suggest that local resolution-associated immune abnormalities can improve after periodontal therapy (93), but whether systemic bone marrow or circulating myeloid imprints recover with the same kinetics remains unknown. Longitudinal immune phenotyping studies are therefore needed to determine whether periodontal treatment restores systemic myeloid homeostasis and whether such recovery lags behind clinical control of periodontal inflammation.

### 4.4 Integrated appraisal: evidence grades and the decisive missing step

Confidence levels differ markedly across the candidate mechanisms discussed above (**Table 2**). The neutrophil–NET axis has relatively strong functional support at both ends of the proposed connection: periodontitis is associated with systemic neutrophil/NET abnormalities and can induce neutrophil priming, whereas NETs have direct pathogenic effects on inflammation and fibrosis in MASH (65, 69, 86). It is therefore one of the best-supported myeloid mechanisms potentially linking periodontitis to MASLD.

**Table 2:** Evidence appraisal of candidate myeloid mechanisms linking periodontitis to MASLD.

Candidate mechanism	Periodontal/systemic evidence	MASLD hepatic evidence	Author interpretation
Neutrophil–NET axis	Circulating NET abnormalities and neutrophil priming are supported by human and experimental studies; periodontitis can induce HSPC neutrophil-lineage bias (65, 69, 89)	NETs activate HSCs and promote fibrosis; NET inhibition attenuates fibrosis in MASH models (86, 87)	The most coherent candidate relay, but the cross-organ step remains unproven. Periodontitis is more likely to modify neutrophil state than to provide liver-directed NETs directly.
Monocyte–macrophage axis	Monocytes are continuously recruited to periodontal lesions, but a stable periodontitis-specific circulating monocyte program has not been established (44, 52, 65, 70)	Monocyte recruitment and niche-dependent MoKC/LAM differentiation are prominent during MASH progression (47, 78, 83, 91)	More likely to reflect how the MASLD liver receives and reshapes systemic myeloid input than a periodontitis-specific relay.
Resolution failure	Impaired efferocytosis and defective pro-resolving programs occur in periodontitis (92, 93)	Defective TREM2-dependent efferocytosis sustains inflammation, whereas TREM2^+^ LAMs support resolution (80, 94)	Best viewed as a shared susceptibility state rather than a transport pathway.

The monocyte–macrophage axis is less symmetrical. On the MASLD side, evidence is strong for peripheral monocyte recruitment, niche-dependent macrophage differentiation, and extensive remodeling of the hepatic macrophage compartment during disease progression (47, 78). Whether periodontitis establishes a stable, traceable, and functionally relevant systemic monocyte program, by contrast, remains insufficiently demonstrated. Resolution failure represents a different type of connection: rather than representing an independent cross-organ transmission pathway, impaired efferocytosis and defective pro-resolving programs may provide a shared state of inflammatory susceptibility in periodontal and hepatic tissues (92, 94).

None of these mechanisms currently constitutes an established periodontitis–myeloid cell–liver–MASLD causal pathway. Direct experimental evidence shows that periodontitis can aggravate MASLD, but does not establish that the effect is mediated by periodontitis-conditioned neutrophils, monocytes, or their progeny (68). Whether a systemic immune alteration translates into hepatic pathology is therefore likely to depend both on the nature of the incoming immune signal and on the disease stage and local immune environment of the recipient liver.

Taken together, the evidence from both ends supports three judgments. First, we propose that periodontitis may act as a chronic, fluctuating systemic immune modifier rather than a discrete “second hit” or initiating cause of MASLD; this interpretation is based on experimental evidence of periodontal inflammation-induced myeloid reprogramming and remains to be tested in relation to MASLD progression (44, 65). Second, disease stage may shape the form of the effect: during the steatosis-to-MASH translation, the liver may represent a more permissive environment for amplification of incoming myeloid signals, whereas once advanced fibrosis is established and a scar-associated microenvironment (recruited macrophages, activated fibroblasts, and endothelial remodeling) is in place (95, 96), periodontal inflammation may modify the rate of progression or the balance between inflammation and repair than to determine whether the liver crosses the threshold into MASH. Third, periodontitis-associated systemic myeloid alterations may intersect with MASH at several nodes: the matching of primed neutrophils with hepatic chemotactic signals, the recruitment of CCR2^+^ monocytes, and the differentiation trajectories of recruited cells within a rewired Kupffer cell niche (47, 65, 79). Similarly, if periodontitis contributes to MASH fibrosis, a more plausible mechanism is that it alters the state of circulating myeloid cells entering the liver rather than acting directly on hepatocytes or HSCs; the periodontitis–NET–HSC step has not been directly demonstrated. These links are mechanistically plausible but require cross-organ tracing and interventional mediation experiments for validation. Analogous cross-organ mechanisms proposed in the comorbidity of periodontitis and chronic kidney disease may provide a supporting parallel for the present model (60). This body of evidence defines the mechanistic credibility of the cross-organ myeloid model, but its clinical relevance must still be tested in human studies. The next section evaluates what observational, longitudinal, genetic, biomarker, and interventional evidence can and cannot support.

## 5 Human evidence: association without demonstrated cross-organ myeloid coupling

### 5.1 Association and temporality: insufficient to establish myeloid mediation

Human studies generally support clinical co-occurrence between periodontitis and steatotic liver phenotypes, although the magnitude of the association varies substantially with periodontal definitions, hepatic outcome measures, study design, and adjustment for metabolic confounders (13, 20, 21, 97–100; **Table 1**). Periodontitis and MASLD share major upstream determinants, including obesity, type 2 diabetes, insulin resistance, smoking, lifestyle factors, and socioeconomic disadvantage. Accordingly, adjustment for metabolic background can markedly attenuate the observed association (20, 97, 100).

This heterogeneity limits direct comparison of effect sizes and contributes to inconsistency across studies. Longitudinal studies provide evidence for temporality but do not resolve causality. Baseline periodontitis or cumulative periodontal tissue destruction has been associated with incident fatty liver disease in some cohorts, whereas greater periodontal attachment loss has been linked to subsequent fibrotic progression, particularly in metabolically susceptible individuals (11, 12, 22). Conversely, NAFLD-related metabolic burden has also been associated with later development of periodontitis (27). These findings are therefore compatible with a complex and potentially bidirectional relationship rather than a simple unidirectional causal pathway.

The heterogeneity of this relationship may also reflect differences in host characteristics across study populations. Age and sex may influence both periodontal inflammatory responses and susceptibility to metabolic liver injury, although whether they modify the periodontal–hepatic relationship or the proposed myeloid pathway remains unclear. Individual studies have reported differences in effect estimates according to sex or metabolic susceptibility, whereas obesity appears to identify a subgroup in which the association between periodontal inflammation and hepatic outcomes may be more evident (22, 25). In addition, alcohol use, smoking, type 2 diabetes, insulin resistance, and obesity represent important shared determinants of both periodontitis and MASLD and may contribute to variability in observed associations (5, 8–10, 20, 97, 100). Therefore, future studies examining the periodontal–myeloid–liver connection should consider these factors not only as adjustment variables but also as potential modifiers of biological susceptibility.

Beyond the temporal ordering between periodontal and hepatic phenotypes, the severity and duration of periodontal inflammation may also be relevant to systemic myeloid alterations. Human studies have reported associations between periodontitis severity and circulating NET-associated abnormalities, suggesting that periodontal inflammatory burden may be linked to systemic neutrophil activation (69). However, these observations do not establish persistent myeloid reprogramming or clarify whether the observed systemic abnormalities depend on the duration of periodontal inflammation. Available longitudinal studies have generally assessed periodontal status at baseline without repeated characterization of inflammatory persistence, recurrence, or cumulative exposure over time (11, 12, 22, 23, 26, 27). Moreover, cumulative periodontal tissue destruction may not fully reflect the duration of inflammatory exposure or current inflammatory activity. Whether prolonged periodontal inflammation is associated with stronger or more durable myeloid reprogramming remains unresolved. Human studies integrating longitudinal periodontal inflammatory burden, circulating myeloid phenotypes, and subsequent hepatic outcomes are still lacking.

Genetic evidence is similarly inconsistent. Bidirectional Mendelian randomization studies have reported positive, null, and limited reverse-direction findings (14–16), and a recent meta-analysis of Mendelian randomization studies likewise showed method-dependent heterogeneity (101). Current epidemiological and genetic evidence therefore does not establish periodontitis as an independent cause of MASLD.

For the disease-modifier model considered here, however, the more relevant question is not simply whether periodontitis predicts incident MASLD, but whether periodontal inflammation modifies the subsequent course of an already metabolically stressed liver. Existing longitudinal studies cannot answer this mechanistic question because they have not serially assessed neutrophil function, NET burden, circulating monocyte phenotypes, or other systemic myeloid intermediates together with hepatic outcomes. They therefore support statistical association and, in some settings, temporal ordering, but not a mechanistic mediation chain of periodontitis → altered myeloid state → hepatic remodeling.

### 5.2 Human myeloid evidence: still compartmentalized between the two diseases

Human studies indicate that periodontitis can alter the systemic inflammatory environment, but commonly measured markers have limited mechanistic resolution. Periodontal treatment can reduce circulating C-reactive protein (CRP), with reductions in IL-6 and other inflammatory mediators reported in some studies (61, 62, 102, 103). Composite indices such as the neutrophil-to-lymphocyte ratio (NLR) have also been associated with both periodontitis and more advanced NAFLD/MASH phenotypes (104, 105). These measures support the presence of systemic inflammation but do not identify the cellular pathway linking periodontal inflammation to the liver.

More mechanistically informative observations are available at the myeloid-cell level. Severe periodontitis is associated with circulating NET complexes, NET-associated modified histones, altered neutrophil ROS responses, and modest changes in circulating monocyte populations (69, 106). On the hepatic side, circulating monocytes from patients with MASH display a distinct immunometabolic phenotype characterized by increased glycolysis and mitochondrial respiration, enhanced complex II activity, and increased ROS-linked inflammatory output (91). These findings show that systemic myeloid abnormalities can be detected in both disease contexts.

The major limitation is that the two ends have rarely been examined within the same individuals. Evidence for periodontal-associated neutrophil or monocyte abnormalities and evidence for MASLD-associated circulating myeloid dysfunction currently arise largely from separate cohorts and experimental settings. Similar cellular phenotypes therefore cannot establish common cellular origin, inter-organ trafficking, or functional coupling.

Direct testing of the proposed model would require an integrated oral–circulating myeloid–liver phenotype in which periodontal inflammatory burden, circulating neutrophil and monocyte states, and hepatic disease activity are measured serially within the same population. At present, human evidence cannot demonstrate that periodontitis-conditioned myeloid cells subsequently participate in remodeling of the hepatic immune compartment in MASLD.

### 5.3 Periodontal treatment as a mechanistic perturbation, not yet a mediation test

Periodontal treatment provides an opportunity to perturb one component of the proposed cross-organ system in humans. In the multicenter PERION randomized controlled trial, 40 patients with NAFLD and periodontitis underwent scaling and root planing or usual care. At 12 weeks, periodontal treatment was accompanied by a greater reduction in ALT and by reductions in serum *Porphyromonas gingivalis* IgG antibody titers; exploratory imaging measures, including MRI-PDFF, MRE, and VCTE-derived indices, also changed during follow-up (29).

The mechanistic significance of PERION lies less in demonstrating a therapeutic effect on MASLD than in showing that periodontal inflammatory burden can be experimentally modified in humans while hepatic surrogate endpoints are measured in parallel. The trial was small and open-label, relied largely on biochemical and noninvasive surrogate outcomes, and did not demonstrate histologic MASH resolution, fibrosis regression, or long-term liver-related clinical benefit (29, 107, 108).

More importantly for the present framework, PERION did not serially determine whether periodontal treatment changed systemic neutrophil function, NET burden, circulating monocyte states, or other myeloid intermediates. It therefore could not test whether a change in systemic myeloid state mediated the observed hepatic changes.

Taken together, current human evidence establishes clinical co-occurrence, provides limited information on temporal direction, and shows that periodontal inflammatory burden can be experimentally perturbed. What remains missing is a human study that serially connects periodontal inflammation → systemic myeloid state → hepatic phenotype within the same individuals.

## 6 Testing the missing bridge

Moving from epidemiological association to mechanism requires study designs that directly test the proposed cross-organ myeloid axis. Four elements deserve priority: selecting biologically informative populations, establishing an integrated oral–immune–liver phenotype, using periodontal treatment as a mechanistic perturbation, and addressing the key causal steps of source, destination, timing, and reversibility.

### 6.1 Lineage and destination: do periodontitis-conditioned myeloid cells reach the MASLD liver?

The first requirement for validating the proposed cross-organ bridge is to establish cellular origin and destination. Similar transcriptional or functional states in periodontal, circulating, and hepatic myeloid populations are insufficient to demonstrate that the same cellular lineage has moved between compartments. Single-cell and spatial multi-omics can define cellular states and niche-specific programs, but cannot by themselves establish inter-organ migration.

Animal studies should therefore directly track myeloid cells generated after periodontal inflammatory conditioning. Fate-mapping approaches, bone marrow transplantation, adoptive transfer, and other lineage-informed strategies could determine whether cells arising from a periodontitis-conditioned hematopoietic compartment enter the steatotic or MASH liver and contribute to its recruited myeloid pool (44, 47, 83). Cell tracking should be coupled to phenotypic analysis after hepatic entry to determine whether the incoming cells retain features of their pre-hepatic state or are rapidly overwritten by the local liver niche.

This distinction is essential. Demonstrating that periodontitis alters circulating myeloid cells does not establish that those cells reach the liver, and detecting similar inflammatory programs in hepatic myeloid populations does not establish a periodontal origin. Direct lineage evidence is therefore the first missing component of the proposed relay.

### 6.2 Stage dependence and necessity: does the recipient liver determine the effect of myeloid input?

Establishing cellular trafficking alone would not demonstrate pathogenic relevance. The next question is whether periodontitis-conditioned myeloid input produces different functional consequences according to the hepatic microenvironment it encounters. The model developed in this review predicts that the recipient liver is not static: simple steatosis provides a metabolically stressed but relatively limited inflammatory niche, the steatosis-to-MASH transition is characterized by increasing chemotactic and innate immune amplification, and established MASH and fibrosis contain extensively remodeled macrophage and stromal niches.

This prediction can be tested experimentally by introducing an equivalent periodontitis-conditioned myeloid input into animals representing different stages of MASLD and comparing cellular recruitment, differentiation, spatial localization, inflammatory activity, and hepatic outcomes. Such experiments would directly test whether the same pre-hepatic myeloid state is interpreted differently by steatotic, inflammatory, and fibrotic hepatic environments.

Functional necessity must also be established. If periodontitis-conditioned neutrophils or monocytes contribute to hepatic disease modification, selective depletion, recruitment blockade, or targeted disruption of relevant effector pathways should attenuate the corresponding hepatic phenotype. Conversely, adoptive transfer of defined periodontitis-conditioned myeloid populations could test whether these cells are sufficient to modify inflammatory or fibrogenic responses in a stage-dependent manner.

The central prediction is therefore not simply that periodontitis generates inflammatory myeloid cells, but that the biological consequence of this altered myeloid input depends on the state of the recipient liver.

### 6.3 Reversibility and mediation: can periodontal treatment serve as a test in humans?

Human validation requires a design that links the three relevant compartments within the same individuals. Mechanistic studies should therefore enrich for populations in whom a periodontal–hepatic interaction is most likely to be detectable, including patients with active MASH or at risk of MASH, clinically relevant fibrosis risk, and a high periodontal inflammatory burden. Obesity, type 2 diabetes, and other major cardiometabolic factors should be prespecified for stratification because they may modify both periodontal inflammation and MASLD progression (5, 22, 107).

Phenotyping should simultaneously capture periodontal inflammatory burden, systemic myeloid state, and hepatic disease activity. Periodontal measures may include staging and grading, bleeding on probing, and PISA (109). Blood assessment should prioritize mechanistically informative myeloid measures, including circulating NET-related markers, neutrophil functional responses, and monocyte phenotypes, while CRP and NLR can provide complementary background information (69, 91). Hepatic assessment should extend beyond transaminases and incorporate quantitative or fibrosis-related measures such as MRI-PDFF, VCTE/MRE, ELF, and histology when clinically available (5, 107).

Periodontal therapy can then be used as a mechanistic perturbation. Future trials should test three sequential levels of response: reduction of periodontal inflammatory burden, alteration of the systemic myeloid state, and subsequent change in hepatic phenotype. Sampling should distinguish the transient inflammatory response that may occur immediately after intensive periodontal therapy from the later resolution of chronic periodontal inflammation (61, 110). An additional consideration is the persistence and reversibility of periodontal inflammation-induced myeloid reprogramming, a concept informed by experimental studies of trained immunity (39, 44, 65–67). Because circulating neutrophils and monocytes are short-lived (51, 63), longitudinal studies should determine whether periodontal treatment normalizes circulating myeloid phenotypes and, where assessable, hematopoietic output, or whether residual trained immune features persist after local inflammatory resolution. Serial assessment before and after periodontal intervention will be required to define the duration, reversibility, and functional relevance of this potential systemic immune memory.

The critical analysis should move beyond simple pre–post comparisons. Longitudinal mediation models should directly test whether periodontal treatment → change in systemic myeloid state → change in hepatic phenotype is supported within the same individuals. Evidence that myeloid changes temporally precede and statistically mediate hepatic improvement would provide substantially stronger support for the proposed cross-organ mechanism than improvement in ALT or liver stiffness alone.

Ultimately, validation of the framework requires three complementary forms of evidence: lineage evidence showing that periodontitis-conditioned myeloid cells reach the MASLD liver, functional evidence showing that these cells are necessary for changes in hepatic inflammation or disease progression, and stage-specific evidence showing that their effects vary according to the hepatic microenvironment they encounter. Human studies must then determine whether reversal of the periodontal inflammatory state modifies systemic myeloid phenotypes and whether those changes mediate hepatic improvement.

## 7 Conclusion and prospects

Current evidence supports both ends of the proposed cross-organ myeloid framework, but the direct connection between them has not yet been established. At the periodontal end, experimental studies show that chronic periodontal inflammation can reprogram bone marrow hematopoietic stem and progenitor cells (HSPCs), alter myeloid output, and maintain neutrophils in a persistently primed state; human studies have also identified systemic NET-associated abnormalities and other signs of myeloid activation. At the hepatic end, MASLD progression is accompanied by increased recruitment of peripheral myeloid cells, disruption and remodeling of the Kupffer cell niche, diversification of monocyte-derived macrophage states, and functional interactions between neutrophil-derived signals and profibrotic pathways. Taken together, these two bodies of evidence provide biological plausibility for a cross-organ myeloid connection, but they do not demonstrate that periodontitis-conditioned myeloid cells actually enter the MASLD liver, much less that they participate in subsequent hepatic immune remodeling.

This missing step is central to whether the framework ultimately holds. The key question is not simply whether periodontitis generates a systemic inflammatory state, but whether myeloid cells altered under conditions of chronic periodontal inflammation can be recruited to the liver and retain sufficient features of their pre-hepatic primed state to influence local immune responses. Even if such cellular trafficking does occur, its biological consequences are unlikely to remain uniform across different stages of MASLD. The recipient liver itself changes substantially during disease progression: in simple steatosis, the liver is exposed to metabolic stress but has a relatively limited capacity for inflammatory amplification; during the transition from steatosis to MASH, chemotactic signaling and innate immune amplification become increasingly prominent; and in established MASH and fibrosis, macrophage and stromal niches are extensively remodeled. If periodontitis-conditioned myeloid cells reach the liver, their recruitment, reshaping, and functional consequences may differ according to the stage of liver disease.

Whether this framework is ultimately validated will depend on obtaining evidence that directly connects these two supported ends. Specifically, lineage-tracing and cell-tracking studies are required to determine whether periodontitis-conditioned myeloid cells actually reach the MASLD liver; functional perturbation studies are needed to establish whether these cells are necessary for changes in hepatic inflammation or fibrosis; and stage-specific experiments should determine whether the same myeloid input produces different consequences in steatotic, MASH, and fibrotic livers. In human studies, periodontal inflammatory burden, circulating myeloid phenotypes, and hepatic outcomes should be longitudinally integrated within the same individuals, with further assessment of whether periodontal treatment alters systemic myeloid states and whether such changes mediate subsequent hepatic improvement. Until these critical steps are directly demonstrated, the “periodontium–bone marrow–circulating myeloid cell–liver” axis should be regarded as a testable mechanistic framework rather than an established causal pathway.
